# Nicotin in Tobacco

**Published:** 1913-01

**Authors:** 


NICOTIN IN TOBACCO. *London Lancet* (April 6th, 1912) By a new method of analysis, the *Lancet* finds a much smaller percentage of nicotin in tobacco than has heretofore been supposed. Havana cigars contain .64% nicotin, British, 1.24%. Virginian and Turkish cigarettes contain 1.38 to 1.60% nicotin, Caporal, 2.60%, and average pipe mixtures 2.85%. The percentage of the nicotin-content of tobacco that goes into the smoke, and is drawn into the mouth is as follows:

"Cigarettes: Virginian 3.75 to 8.50%; Turkish 37%; Caporal 84%; pipe-mixtures, smoked in cigarettes, 79%; pipes 77 to 92%; cigars 31 to 83%."

